# Empowering the willing: the feasibility of tele-mentored self-performed pleural ultrasound assessment for the surveillance of lung health

**DOI:** 10.1186/s13089-021-00250-6

**Published:** 2022-01-03

**Authors:** Andrew W. Kirkpatrick, Jessica L. McKee, Chad G. Ball, Irene W. Y. Ma, Lawrence A. Melniker

**Affiliations:** 1 TeleMentored Ultrasound Supported Medical Interventions (TMUSMI) Research Group, University of Calgary, Calgary, AB Canada; 2 Departments of Surgery, University of Calgary, Calgary, AB Canada; 3 Departments of Critical Care Medicine, University of Calgary, Calgary, AB Canada; 4 Regional Trauma Services, EG 23, Foothills Medical Centre, 1403 29 St NW, Calgary, AB T2N 2T9 Canada; 5 Canadian Forces Medical Services, University of Calgary, Calgary, AB Canada; 6 W21C, University of Calgary, Calgary, AB Canada; 7 John A. Buchanan Chair, Division of General Internal Medicine, University of Calgary, Calgary, AB Canada; 8 Department of Emergency Medicine, New York-Presbyterian Brooklyn Methodist Hospital, New York, NY USA

**Keywords:** COVID-19, Telemedicine, Lung Sonography, Remote Telementoring, Community Health, SARS-CoV-2, Patient focused health-care

## Abstract

**Background:**

SARS-CoV-2 infection, manifesting as COVID-19 pneumonia, constitutes a global pandemic that is disrupting health-care systems. Most patients who are infected are asymptomatic/pauci-symptomatic can safely self-isolate at home. However, even previously healthy individuals can deteriorate rapidly with life-threatening respiratory failure characterized by disproportionate hypoxemic failure compared to symptoms. Ultrasound findings have been proposed as an early indicator of progression to severe disease. Furthermore, ultrasound is a safe imaging modality that can be performed by novice users remotely guided by experts. We thus examined the feasibility of utilizing common household informatic-technologies to facilitate self-performed lung ultrasound.

**Methods:**

A lung ultrasound expert remotely mentored and guided participants to image their own chests with a hand-held ultrasound transducer. The results were evaluated in real time by the mentor, and independently scored by three independent experts [planned a priori]. The primary outcomes were feasibility in obtaining good-quality interpretable images from each anatomic location recommended for COVID-19 diagnosis.

**Results:**

Twenty-seven adults volunteered. All could be guided to obtain images of the pleura of the 8 anterior and lateral lung zones (216/216 attempts). These images were rated as interpretable by the 3 experts in 99.8% (647/648) of reviews. Fully imaging one’s posterior region was harder; only 108/162 (66%) of image acquisitions was possible. Of these, 99.3% of images were interpretable in blinded evaluations. However, 52/54 (96%) of participants could image their lower posterior lung bases, where COVID-19 is most common, with 99.3% rated as interpretable.

**Conclusions:**

Ultrasound-novice adults at risk for COVID-19 deterioration can be successfully mentored using freely available software and low-cost ultrasound devices to provide meaningful lung ultrasound surveillance of themselves that could potentially stratify asymptomatic/paucisymptomatic patients with early risk factors for serious disease. Further studies examining practical logistics should be conducted.

*Trial Registration*: ID ISRCTN/77929274 on 07/03/2015.

**Supplementary Information:**

The online version contains supplementary material available at 10.1186/s13089-021-00250-6.

## Introduction

COVID-19 pneumonia is disrupting life on the planet earth in an unprecedented fashion. While many if not most people have mild or asymptomatic disease, others, even young previously healthy people, may become rapidly sick with severe hypoxia despite exhibiting minimal symptoms, including dyspnea [1–3]. The burden on health care systems may be extraordinary, with even well-developed nations’ health care systems being overwhelmed. Health care providers may be particularly susceptible if appropriate infection prevention and control measures are not in place [4]. Thus, solutions need to be sought to provide excellent patient care, but also to protect provider health. COVID-19 is a paradox, as despite the risk to providers, the majority contracting the virus will not develop COVID-19 pneumonia. Most will have none or minor symptoms and can safely self-isolate at home. However, those who develop severe disease, need to be identified early [4].

Compared to chest radiographs and computed tomography (CT), lung ultrasound (LUS) is a simpler, more portable, economical, and potentially home-based technology that might be used for at-risk patients to self-monitor their lungs of for early signs of COVID-19 pneumonia [5–7]. Findings from COVID-19 pneumonia are typically present in the lung periphery [8–10], an anatomic fact that permits LUS to be used to diagnose and manage all phases of care in COVID-19 [5, 9, 11]. LUS may detect early disease progression as the lungs deteriorate from normal to an alveolar-interstitial pattern of lung disease with single discrete vertical artifacts (B-lines) or confluent B-lines [9, 11]. Through work onboard the International Space Station examining self-performed telementored lung ultrasonography (SPTMLUS) performed by inexperienced point-of-care users guided by remote experts [12], we have long known that accurate ultrasound images of the lungs can be self-obtained [13–17]. What has never been examined, is whether willing but ultrasound-naïve adults can be remotely mentored to obtain meaningful lung ultrasound images upon themselves to triage alveolar-interstitial pneumonic diseases, such as COVID-19. The purpose of this study was thus to examine the feasibility and quality of SPTMLUS of novices when expertly guided. Furthermore, this paradigm may be considered a specific example of a broader concept that may contribute to many facets of patient focused and individualized healthcare.

## Methods

This study was registered and ethically approved and structured to comply with the SQUIRE reporting guidelines [18]. A healthy cohort of self-isolating participants in Edmonton, Alberta conducted SPTMLUS examinations mentored by a remote expert. The participants were self-isolating among their family units in response to applicable Public Health orders in effect. After informed consent, participants completed an electronic demographic survey (Additional file 1) and received a package containing a disinfected hand-held high-frequency linear ultrasound probe (Philips Lumify, Philips, Amsterdam, NL), and a package of sterile ultrasound-gel. They also watched a brief instructional video on how to hold the probe and where anatomically they would be guided to scan (Additional file 2).

**Fig. 1:**
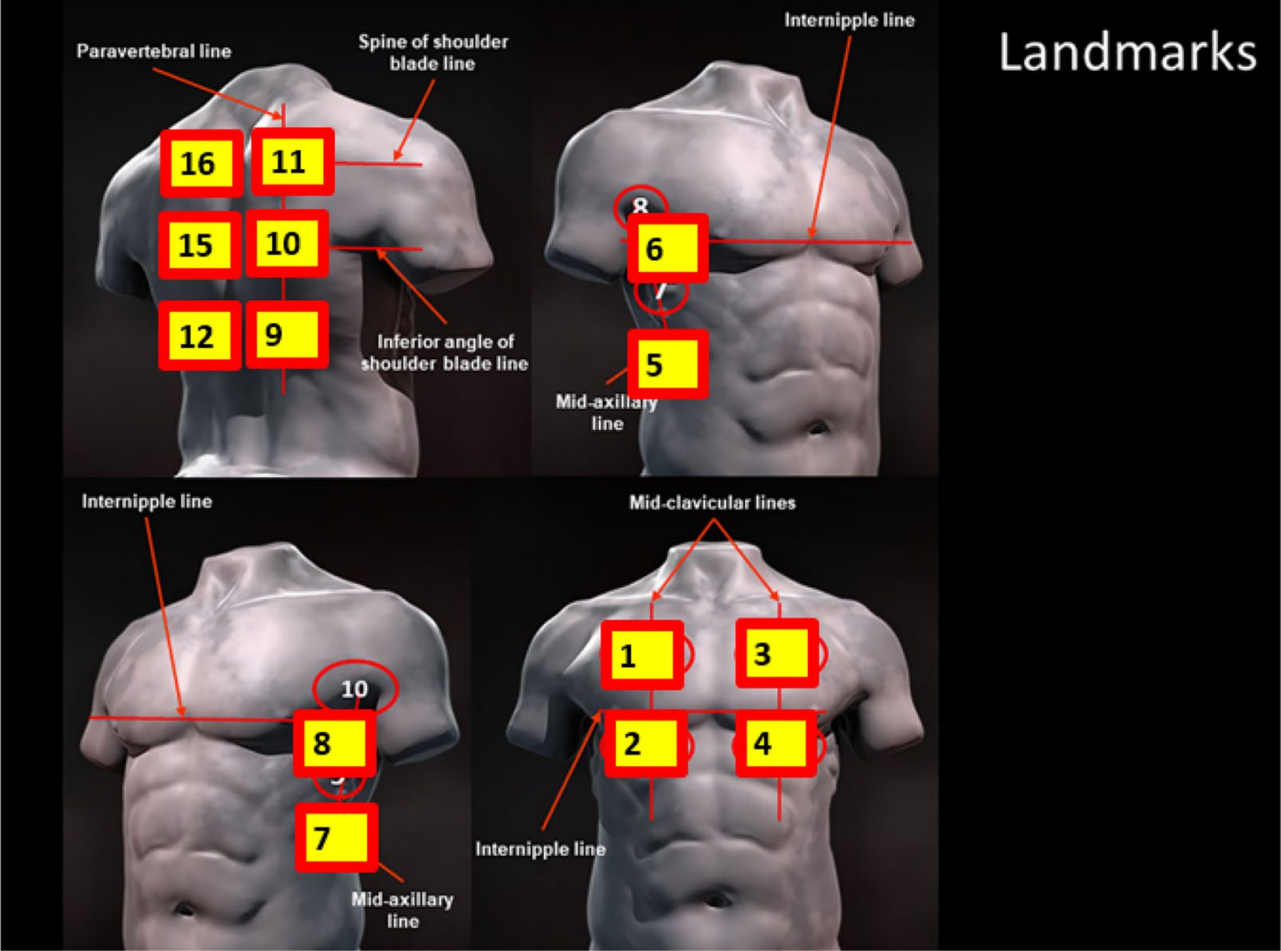
Anatomic locations targeted for the self-performed lung examination. Figure [19] modified from proposal for international standardization of the use of lung ultrasound for patients with COVID‐19 by Soldati et al., J Ultrasound Med 2020, published in Open Access Format

**Fig. 2:**
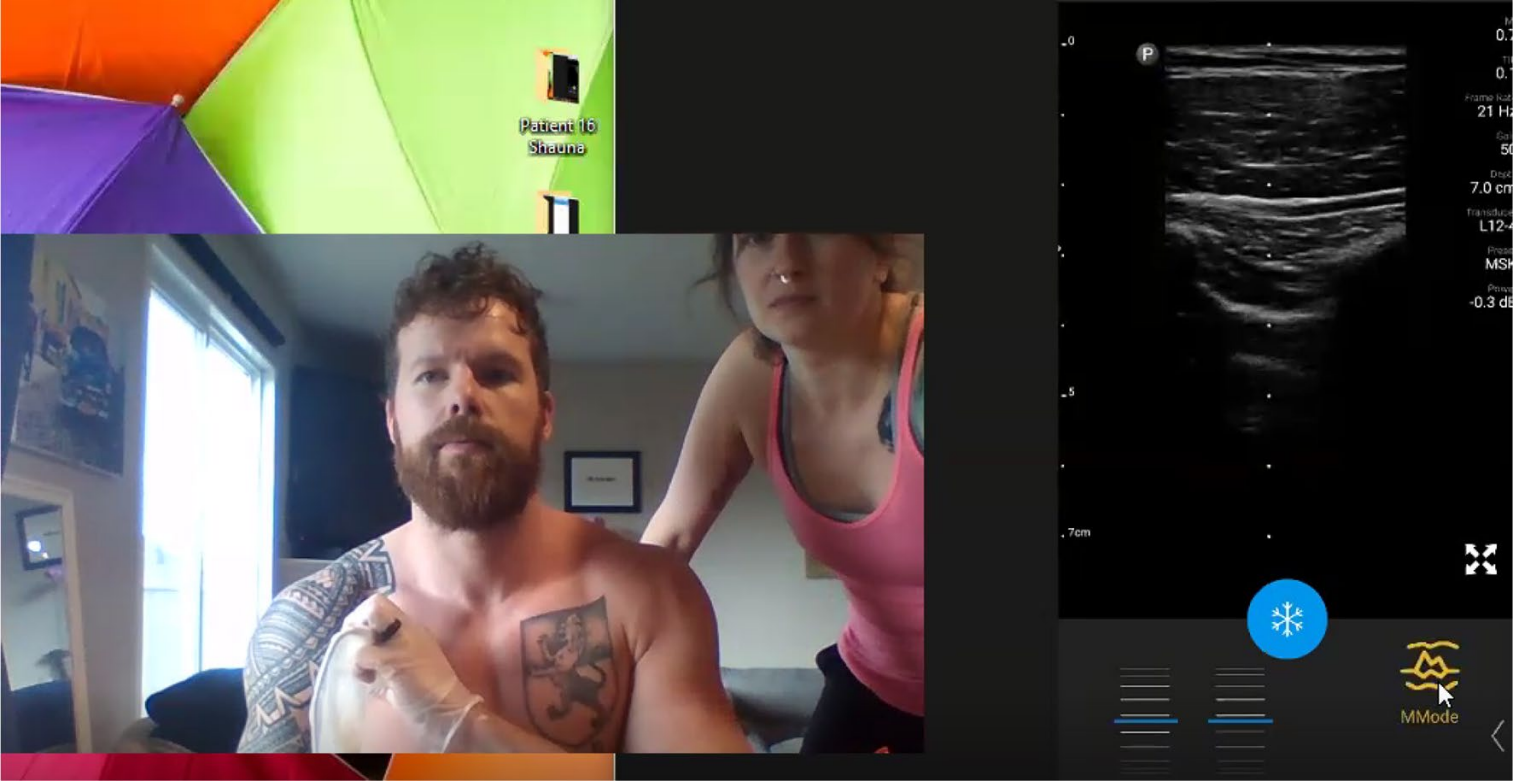
Representative still image of mentee self-performing lung ultrasound to demonstrate the pleural interface. Ultrasound Naïve participant being instructed to demonstrate the pleural interface of the visceral and parietal pleural illustrating the “Batwing” sign of Lichtenstein [20]. It should be noted that all lung ultrasound is a dynamic examination better viewed real time and in video recordings (Additional file 4) than still images

**Fig. 3:**
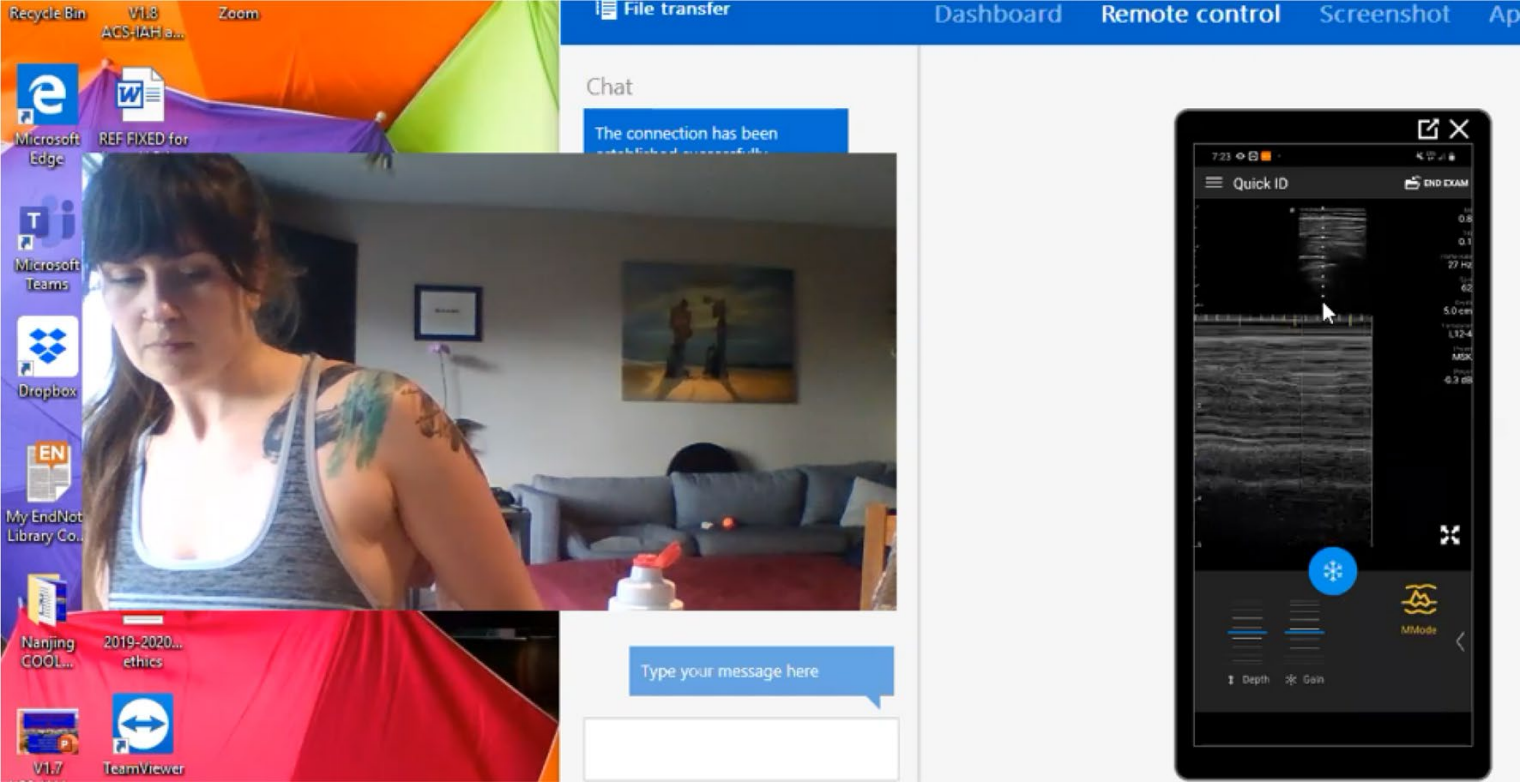
Representative still image of mentee self-performing lung ultrasound to demonstrate the pleural interface of her back. Ultrasound Naïve participant being instructed to demonstrate the pleural interface of the visceral and parietal pleural illustrating the “Seahorse” sign of Lichtenstein [20]. It should be noted that all lung ultrasound is a dynamic examination better viewed real time and in video recordings (Additional file 3) than still images

**Fig. 4:**
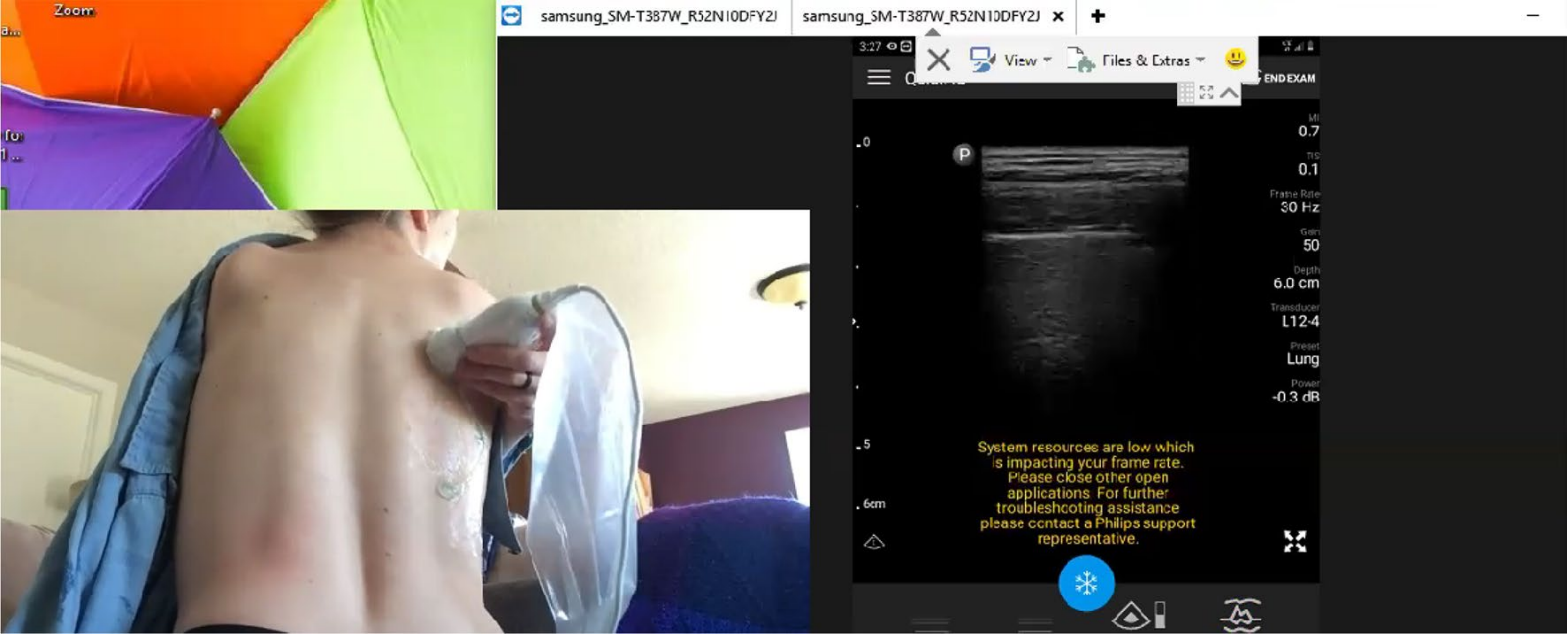
Representative still image of mentee self-performing lung ultrasound to demonstrate the pleural interface of her back. It should be noted that all lung ultrasound is a dynamic examination better viewed real time and in video recordings (Additional file 3) than still images

Thereafter, a lung ultrasound expert in Calgary (AWK) guided the subjects to measure their blood pressure (details reported elsewhere) and conduct a standardized lung examination, using Zoom Teleconferencing (Zoom, Hillsboro, OH). The desired examination was based on the 14-zone method proposed by Soldati for International Standardization of the Use of Lung Ultrasound for Patients with COVID-19 [19] (Fig. 1). The study goal was to generate an adequate “Batwing” depiction of the pleura interface between two rib shadows at each location on the thorax [20] (Fig. 2). Outcome measures were, therefore, (1) whether the subject was physically able to reach all 14 desired anatomic locations (Figs. 3 and 4) and (2) whether the quality of images was considered “adequate” for image interpretation and diagnosis. Images were optimized through remote control of ultrasound “knobology” by the mentor using remote access software (Teamviwer, Göppingen, Germany). The pleural interface was interrogated with 2D, M-mode, and color-Power Doppler (CPD) modes[21], and all image acquisition attempts were videorecorded. The mentor scored each of the pleural images real time using the proposed Soldati method from 0 (normal) to 3 (very abnormal) (Additional file 4) [19], and counted the number of B-lines present at each anatomic location. Each participant completed an online post-test evaluation that included their perceptions of the difficulty in performing their self-examination including a 5-point Likert scale rating the examination at each location as being one of; 1—Very Hard, 2—Hard, 3—Neutral, 4—Easy, 5—Very Easy (Additional file 3).

**Fig. 5:**
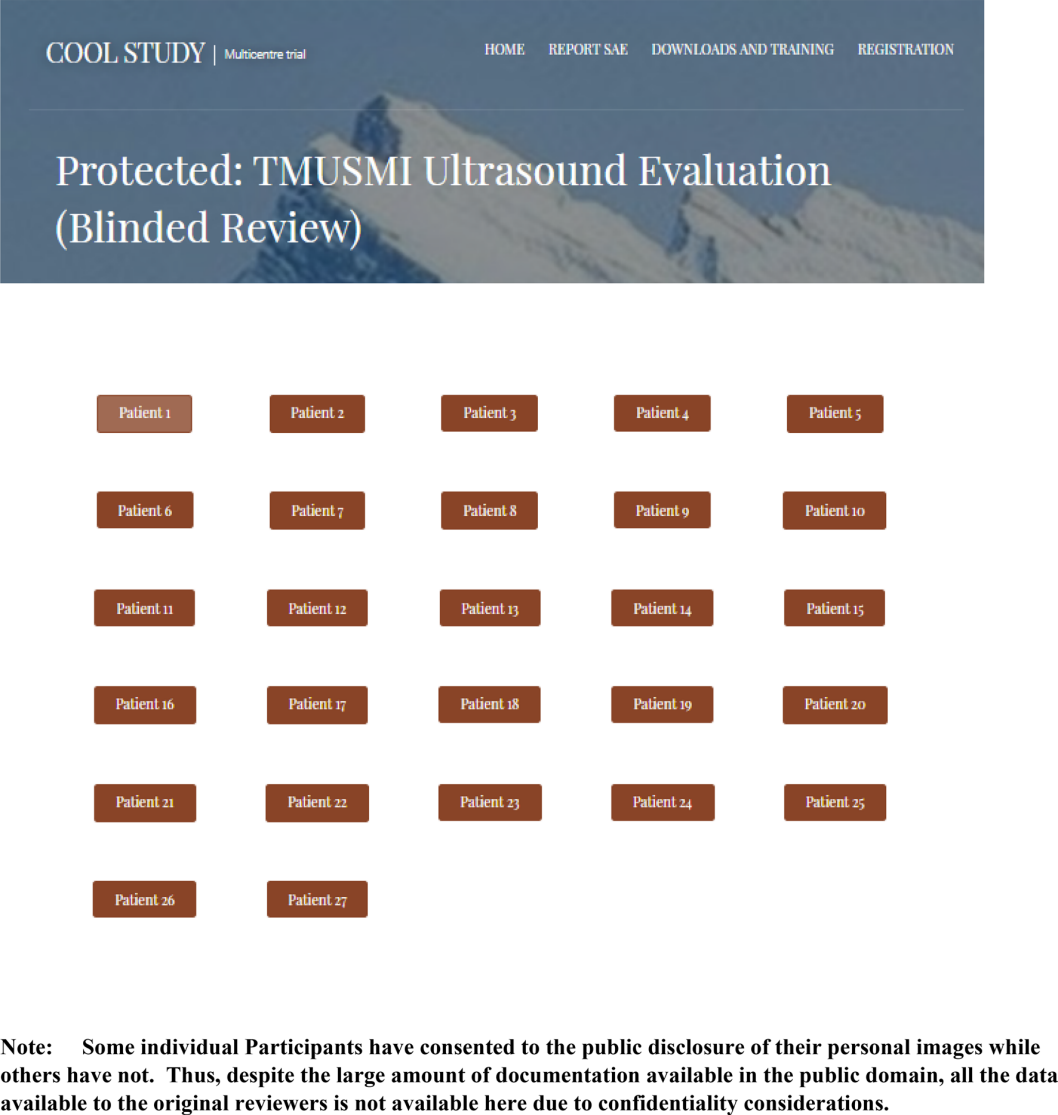
Webpage of selected still images and full video recordings of each examination for blinded review. Some individual Participants have consented to the public disclosure of their personal images, while others have not. Thus, despite the large amount of documentation available in the public domain, all the data available to the original reviewers is not available here due to confidentiality considerations. Videorecording of the complete examinations for those subjects who agree to disclose their personal images are available in Additional file 3

Subsequently the mentor retrospectively reviewed all examinations and prepared representative still images and videos at each anatomic location, which were uploaded to a protected website for an a-priori planned independent review by three outside lung ultrasound experts, who rated both the image quality (as adequate vs. inadequate) and the degree of abnormality using the same Soldati scoring protocol[22]. If they had any concerns regarding image quality, they were encouraged to review the entire source examination. Standard descriptive statistics were used. Patients reporting and not reporting any upper body musculoskeletal concerns or shoulder injuries were analyzed separately and the comparisons between groups were made using Fisher’s exact tests. The theoretical ability to complete commonly used and recommended lung ultrasound imaging protocols was calculated by analyzing the ability for SPTMLUS to be completed at each recommended location of the Extended Focused Assessment with Sonography for Trauma [23], the Bedside Lung Ultrasound Assessment [24], and the International evidence-based recommendation for point-of-care lung ultrasound [25], and a theoretical examination just looking at the anterior, lateral, and posterior lung bases (Fig. 5).

## Results

### Demographics

The study population consisted of a convenience sample of 27 self-isolating inhabitants in Edmonton, Alberta, Canada. All participants stated they were comfortable with typical informatic tools of modern society, such as smart-phones and tablet computers, but none were informatic technology experts. Three [11.1%] had prior ultrasound exposure but none had ever performed LUS (Table 1). Fifteen [14.8%) of participants had a Masters or higher Education degree, 7 [26%] reported an upper body musculoskeletal problem and 8 [29.6%] reported a previous shoulder injury (Table 1).

**Table 1:** Demographic profile of the 27 ultrasound-novice self-isolating participants

Demographics	Response (standard deviation) [percentage]
Age	42.81 years (2.33)
Male/Female	15 [56%]/12 [44%]
Height	172.9 cm (10.20)
Weight	80.67 kg (17.82)
BMI	27.0
Highest level of education	
Did not complete High School	2 [7.4%]
High school	4 [14.8%]
Diploma after high school	8 [29.6%]
Trade certificate	1 [3.7%]
Undergraduate degree	7 [25.9%]
Master’s degree	4 [14.8%]
Other	1 [3.7%]
Ever held an ultrasound?	
Regularly	0 [0.0%]
Once^1^	3 [11.1%]
Never	24 [88.9%]
Pre-existing respiratory concerns?	
Yes^2^	4 [14.8%]
No	23 [85.2%]
Pre-existing cardiac concerns	
Cardiac bypass	2 [7.4%]
Premature atrial contractions	1 [3.7%]
None	24 [88.9%]
Smoking or vaping	
Cannabis only	2 [7.4%]
Tobacco only	1 [3.7%]
None	24 [88.9%]
Upper body musculoskeletal problems	
None	20 [74.1%]
Present	7 [25.9%]
History of shoulder injury	
Yes	8 [29.6%]
No	19 [70.4%]
Asthma diagnosis	
Yes	4 [14.8%]
No	23 [85.2%]

^1^Three subjects replied affirmative, none had formal training; one had unofficially been shown Obstetrical ultrasound in Uganda, one Firefighter had previously participated in just-in-time mentored abdominal and vascular ultrasound; one research coordinator had been exposed to an ultrasound phantom: ^2^all reported pre-existing respiratory concerns were self-reported asthma

### Ability to complete a comprehensive pleural ultrasound examination

Overall, all 27 participants were able to obtain images from all (100%) of the eight anatomic locations on the anterolateral chest. The back was relatively harder, although possible on the lower back in 52/54 (96.3%) of attempts; 38/54 (70.4%) midback, and only18/54 (33%) on the upper back (Table 2). When asked the subjective difficulty in performing the examination, participants rated imaging their own anterior and lateral chest as between “easy” and “very easy” in all locations, while the back was rated between “neutral” to “easy” in all locations (Table 3). There was no statistical relation between the ability/inability to obtain images and a history of shoulder injury at any of the anatomic location.

**Table 2:** Aggregate ability to obtain images from the complete thorax and the assessed quality of the images that could be obtained

Anatomic Location	Image generation deemed possible^a^	Image quality deemed adequate (real time mentor)^b^	Image quality deemed adequate (independent review)^c^
(1) Right upper chest	100%	100%	100%
(2) Right lower chest	100%	100%	100%
(3) Left upper chest	100%	100%	100%
(4) Left lower chest	100%	100%	98.8%
(5) Right side lower	100%	100%	100%
(6) Right side upper	100%	100%	100%
(7) Left side lower	100%	100%	100%
(8) Left side upper	100%	100%	100%
(9) Right back lower	100%	100%	98.7%
(10) Right back middle	74.1%	95.2%	98.4%
(11) Right back upper	70.4%	100%	100%
(12) Left back lower	100%	100%	100%
(15) Left back middle	66.7%	100%	100%
(16) Left back upper	37%	100%	100%

Anatomic location is visually demonstrated in Fig. 1

^a^Image Generation was the remote mentoring expert’s assessment of the interaction with the participant as to whether assessing the anatomic location was possible to image

^b^Image Quality was assessed real time by the Mentor during the examination

^c^Image Quality was assessed as the composite result of the a-prior independent review of the images/videos

**Table 3:** Participants subjective post-test assessment of difficulty in providing self-administered ultrasound examination

Anatomic	Median	Mean Self-Reported Score^1^
Location	Rating	(95% confidence interval)
(1) Right Upper Chest	5	4.67 (4.24–5.09)
(2) Right Lower Chest	5	4.67 (4.24–5.09)
(3) Left Upper Chest	5	4.67 (4.24–5.09)
(4) Left Lower Chest	5	4.67 (4.24–5.09)
(5) Right Side Lower	5	4.52 (4.07–4.96)
(6) Right Side Upper	5	4.44 (4.04–4.85)
(7) Left Side Lower	5	4.52 (4.07–4.96)
(8) Left Side Upper	5	4.44 (4.04–4.85)
(9) Right Back Lower	4	3.89 (3.49–4.29)
(10) Right Back Middle	3.5	3.41 (3.01–3.81)
(11) Right Back Upper	3	2.93 (2.41–3.44)
(12) Left Back Lower	4	3.96 (3.55–4.38)
(15) Left Back Middle	4	3.35 (2.89–3.80)
(16) Left Back Upper	3.5	2.85 (2.33–3.37)

Anatomic location is visually demonstrated in Fig. 1

Mean Self-Reported Score^1^ was recorded as a 5-point Likert scale from 1 Hard to 5 Easy (1—Very Hard, 2—Hard, 3—Neutral, 4—Easy, 5—Very Easy)

### Real-time subjective mentored evaluation and a-priori independent review

Although not all desired locations in all participants could be imaged, of the sites that could, the image quality of 322/324 (99.7%) of real-time determinations was felt to be adequate in real time assessment and adequate in 1124/1128 (99.6%) of independent reviews (Table 2). An examination of suspected reasons for inadequate images is presented in Table 4. Results regarding evaluated lung score and B-line counts are available (Additional file 5), recognizing that no patient had known COVID-19 during examination.

**Table 4:** Suspected reasons for inadequate quality images

Anatomic Location	Reason for Image Inadequacy
Left lower anterior chest	Previous median sternotomy with pericardiotomy and cardiac surgery
Right lower back	Technical issue where patient video image obscured the ultrasound image on the recorded video
Right middle back	Technical issue where patient video image obscured the ultrasound image on the recorded video

### Selected standardized lung ultrasound performance difficulty

The theoretical feasibility and participant-rated ease of performance of commonly used LUS protocols is presented in Table 5. The Extended Focused Assessment with Sonography for Trauma (EFAST) examination [23] was 100% feasible and would be “very easy” (mean Likert score 4.67 out of 5) as assessed by the participants, as was the Bedside Lung Ultrasound in Emergency (BLUE), (feasibility 100%; ease of performance score 4.56) [20]. Similarly, the International evidence-based recommendations for point-of care lung ultrasound (ICC-LUS) was 100% feasible and easy (mean score 4.56) [26]. Even the overall Soldati COVID examination was feasible in 85.7% of locations and overall rated easy (mean score 4.07). A new theoretical examination involving the lowermost lung fields of the anterior, lateral, and posterior lung bases would be 98.8% feasible and easy to very easy (mean score 4.37) (Table 5).

**Table 5:** Average naïve mentee scores for selected lung ultrasound protocol performance feasibility and difficulty

Lung ultrasound protocol	Anatomic locations	Feasibility	Average self-rated naïve user score
EFAST examination	1,2,3,4	108/108 (100%)	4.67 (Very Easy)
BLUE examination	1,2,3,4,5,6,7,8	216/216 (100%)	4.56 (Very Easy)
Soldati COVID Examination	1,2,3,4,5,6,7,8, 9,10,11,12,15, 16	324/378 (85.7%)	4.07 (Easy)
ICC-LUS Examination	1,2,3,4,5,6,7,8	216/216 (100%)	4.56 (Very Easy)
Lower Lung Fields	2,4,5,7,9,12	160/162 (98.8%)	4.37 (Easy—Very Easy)

*EFAST* Extended Focused Assessment with Sonography for Trauma [23], *BLUE* Bedside Lung Ultrasound Assessment[44], *Soldati COVID examination* Proposal for International Standardization of the Use of Lung Ultrasound for Patients With COVID-19[22], *ICC-LUS Examination* International evidence-based recommendations for point-of-care lung ultrasound[26]. Mean Self-Reported Score was recorded as a 5-point Likert scale from 1 Hard to 5 Easy (1—Very Hard, 2—Hard, 3—Neutral, 4—Easy, 5—Very Easy)

## Discussion

Our results demonstrate that adults without prior ultrasound experience were remarkably adept at accurately imaging their own chests to generate clinically meaningful images under the guidance of a remote mentor. Every participant was able to satisfactorily image their anterior, lateral, and lower posterior thoracic areas. With the exception of one participant who later contracted COVID-19 [27], all participants were at risk, but remained clinically well. In our opinion, the most important interpretation of these results, beyond the implications to assist with the surveillance of COVID-19, is the potential to explore the paradigm of mentored self-care to other conditions involving remote, self-isolated, or vulnerable populations to better enable and empower their own health maintenance. Furthermore, although the technique required an economical hand-held ultrasound device, all other communication technologies are widely available in many if not most homes currently, thus minimizing costs and maximizing potential opportunities.

Challenges related to COVID-19 include the high infectivity of the virus, rapid mutations of more aggressive variants, the frequency of asymptomatic carriers, and the fact that pauci-symptomatic patients may shed the most virus immediately before exhibiting any symptoms. This makes in-person medical assessments a potentially dangerous undertaking, and one that contributed to the near collapse of many healthcare systems [28, 29]. Furthermore, COVID-19 is predicted to be just one of many future zoonotic-based pandemics that will afflict humans in the future [30]. Thus, early experiences with nearly overwhelmed health systems, prompted recommendations to employ telemedical capabilities to provide advanced outreach capabilities, for the “entire population not only for hospitals” [31]. Such an approach would hospitalize only those with severe disease, with asymptomatic or paucisymptomatic observed at home, thereby decreasing contagion and preserving personal protective equipment [8, 31]. Such an approach necessitates the ability to quickly recognize those who exhibiting signs of deterioration, and “rescuing” them quickly [1, 8]. Previous work has demonstrated that with remote guidance, non-expert point of care providers, who may be as inexperienced as children, can be guided to place an inexpensive ultrasound probe onto the chest to assess the visceral–parietal pleural surface [17].

LUS, a relatively new discipline based on the science of artifact analysis, is now established in emergency and resuscitative medicine [26]. We and others recognize its near unlimited value to manage the COVID-19 crisis [11, 19, 32]. The gold standard diagnostic test for COVID-19 diagnosis is the identification of viral nucleic acids (PCR). However, compared to PCR, CT scan may show disease at an earlier time frame [33], as may LUS [34], even in cases, where the PCR was initially negative [35]. As lung ultrasound is a technology that may diagnosis COVID-19 earlier than PCR testing, is portable and able to go to the patient, relatively economical, can discern the presence of progression of COVID-19 pneumonia, and is easily repeatable over the projected time course of disease, lung ultrasound may be beneficial to follow pleural health over the complete evolution of disease [11]. Through marrying the ability to perform ultrasound upon oneself using remote guidance, all these potential uses might be provided without ever requiring a physical encounter with a health-care provider to completely reduce health care exposure to infection during the initial assessment and risk stratification of healthy but at-risk individuals.

In health, the pleural interface of the normal lung of a healthy human, is typically the only part of the lung that can be viewed with ultrasound [36], as air has the highest acoustic density of any other component of the human body, effectively preventing transmission of ultrasound waves beyond the pleura. Thus, when examining the interface of the parietal pleura of the chest wall and the visceral pleura of the lung, only this interface can be seen. When the lung becomes diseased with COVID pneumonia it has been observed that abnormal lung artifacts arise at the pleural junction [11]. Of note, the patchy bilateral, multifocal ground-glass opacity and abnormalities typically found on CT associated with COVID-19 are predominantly identified in the lower [9, 37, 38] and posterior lung zones [9, 39], the so called COVID hot spots. At the bedside, a critical “tipping-point” for concern stratifying those with home-manageable minor diseases from potentially severe deterioration may be an evolution from the normal A-line pattern of health to a progressively abnormal alveolar-interstitial pattern of lung disease illustrated by initially single or discernable B line artifacts, progressing to confluent vertical artifacts (B-lines) or confluent vertical artifacts, ultimately culminating with white lungs and/or consolidated effusions of severe COVID-19 [11].

Although standard examinations have been proposed [9, 22], there is no single currently accepted “standard” examination. It was not unexpected that participants would be less adept at examining their backs, and a posterior examination does not comprise part of many common lung ultrasound protocols (Table 5). Nonetheless, 96.3% of all participants could image their own lower back, higher than was originally expected, and emphasizing the remarkable abilities of motivated laypersons when mentored.

COVID-19 may be particularly treacherous as there may be profound disassociation between the severity of hypoxemia and preservation of respiratory muscle mechanics, lung compliance, and an absence of dyspnea [40]. Thus, there are patients with COVID-19 who exhibit oxygen levels incompatible with life without dyspnea, sometimes termed “happy hypoxia” but is more precisely termed silent hypoxemia [2]. There are also more than a few limitations of pulse oximetry such as O_2_ Saturation monitoring may not be accurate at very low PaO_2_ [2]. This condition may have an alarming frequency. Busana noted that among patients presenting to hospital with hypoxia consistent with acute respiratory failure, one-third were not dyspneic, including 18% with severely abnormal PaO_2/_FiO_2_ ratios of between 50 and 150, and overall, these patients had a mortality rate of 17.6% [1]. Thus, lung ultrasound offers a technique to potentially detect these patients at the earliest signs of lung swelling. Opportunities for future research include formal statistical comparisons of test performance characteristics of each of these modalities individually. However, we suspect that in actual clinical practice, these modalities would be complementary when used together. Recent guidelines for the use of Lung Ultrasound in managing COVID-19 did NOT recommend serial LUS re-examinations despite its potential utility in managing the patient’s status due to concerns regarding infection transmission risks [9]. However, with the technique of RTMSPLUS these risks are completely obviated. Furthermore, these same guidelines, recommend that LUS should, however, be the initial lung imaging modality of choice in patients with minimal symptoms as LUS has higher sensitivity and lower radiation risks [9]. We have also demonstrated unmanned arial vehicle (drone) delivery of RTMSPLUS, with unlimited potential to access remote and disadvantaged populations [41].

### Pneumothoraces, posterior lung fields, and the lung bases

Although the additional modalities of M-Mode and CPD are not necessarily critical to the inference of COVID-19, they contribute to the diagnosis of pneumothorax which recent guidelines recommend assessing for during a COVID-19 lung ultrasound examination [9]. Recent guidelines also strongly recommend that in addition to the usual anterolateral lungs, that posterior lung zones should be scanned whenever possible [9]. This is an aspect, wherein the healthier asymptomatic/Paucisymptomatic patients are advantaged in not being bedbound and supine and were able to image their lower posterior lung fields 100% of the time.

A potential limitation of our study was that our participants were not sick and were feeling well. Therefore, the majority of the LUS images were normal. However, this is appropriate for a screening test intended for asymptomatic/pauci-symptomatic populations. Our participants were relatively young and nearly all completed high school. Thus, our results may not generalize to an older, or less educated population. Our participants all had access to a computer and internet. Remote guidance may be more challenging in situations, where internet is not available, although we agree that internet availability should be a basic human right [42]. Three participants had previous ultrasound exposure, but none had examined the pleura before. In general males were less able to image their complete backs than females, but this may be less critical for lung surveillance in COVID-19, given the propensity for early COVID to affect the lung bases. Therefore, it may be more important for RTMSPLUS to examine the lung bases (anterior, lateral, and posterior locations) and in our study, the ability to image these locations was 100% (Table 2).

This study constitutes a proof of concept that we hope generates further discussion and analysis of the capabilities, logistical requirements, and human factors challenges in assisting in remotely mentored self-care. Clearly innumerable details require study before such a paradigm is ready for clinical application. Given these practicalities, however, we contend that the concept potentially empowers those remote from fixed hospital care to empower their own healthcare through permitting required imaging of their own anatomy and physiology wherever they are geographically as long as they are connected. Just as necessity drove space medicine to consider innumerable innovative ways to incorporate remotely mentored ultrasound into care paradigms [43], just-in-time mentoring of the isolated may have innumerable applications that deserve examination.

## Conclusions

We contend that providing home-SPRTLUS may be a useful method to provide surveillance of at-risk populations. Besides earlier diagnosis and rescue of severe cases, we postulate that such a proactive approach empowering the willing to manage their own health might also potentially reduce anxiety and increase the “connectedness” of patients and health care providers, an intangible commodity that is severely threatened in these times of strict self-isolation.

## Supplementary Information

**Additional file 1**. Subject pre-test evaluation form.

**Additional file 2**. Pre-examination Introductory Video.

**Additional file 3**. Complete video recordings of examinations. **Additional file 3a**.

**Additional file 4**. Pleural Scoring System for COVID 19.

**Additional file 5**. Mentors and Blinded Reviewers Assessment of Pleural Lung Health Scoring.

## Data Availability

The data sets used and/or analysed during the current study are available from the corresponding author on reasonable request.
