# Correction: Empowering the willing: the feasibility of tele-mentored self-performed pleural ultrasound assessment for the surveillance of lung health

**DOI:** 10.1186/s13089-023-00316-7

**Published:** 2023-05-04

**Authors:** Andrew W. Kirkpatrick, Jessica L. McKee, Chad G. Ball, Irene W. Y. Ma, Lawrence A. Melniker

**Affiliations:** 1 TeleMentored Ultrasound Supported Medical Interventions (TMUSMI) Research Group, University of Calgary, Calgary, AB Canada; 2 Departments of Surgery, University of Calgary, Calgary, AB Canada; 3 Departments of Critical Care Medicine, University of Calgary, Calgary, AB Canada; 4 Regional Trauma Services, EG 23, Foothills Medical Centre, 1403 29 St NW, Calgary, AB T2N 2T9 Canada; 5 Canadian Forces Medical Services, University of Calgary, Calgary, AB Canada; 6 W21C, University of Calgary, Calgary, AB Canada; 7 John A. Buchanan Chair, Division of General Internal Medicine, University of Calgary, Calgary, AB Canada; 8 Department of Emergency Medicine, New York-Presbyterian Brooklyn Methodist Hospital, New York, NY USA

**Correction:** **The Ultrasound Journal (2021) 14:2 **10.1186/s13089-021-00250-6

After publication of the original article [1], concerns have been raised that the trial described in this article does not match the Trial Registration record (ISRCTN77929274) provided in the article. Authors explained how their research evolved over time and have provided evidence of ethics approval that matches the research described in the article. On advice from the Editor, the authors have retrospectively registered this trial with the correct details and description under the following number: ISRCTN14491490.

The original article [1] has been updated.
