# Supplementary material for: Empowering the willing: the feasibility of tele-mentored self-performed pleural ultrasound assessment for the surveillance of lung health
Source: Ultrasound J. 2022 Jan 3;14:2. doi: 10.1186/s13089-021-00250-6 (PMC9417136; doi:10.1186/s13089-021-00250-6)
Supplement: Supplementary file 1 — Additional file 1. Subject pre-test evaluation form. [file 13089_2021_250_MOESM1_ESM.docx]

**Supplementary Material 1. Subject Pre-Test Evaluation Form**

**Subject Number _________**

Age _____ (years)

**Questions**

1. Have you ever held an ultrasound probe before?

_____ No _____ Once _____ I use ultrasound regularly?

1. Do you have any known respiratory (breathing) problems?

_____ No ______ Yes (If so describe ________________________)

1. Do you have any known cardiac (heart) problems?

_____ No ______ Yes (If so describe ________________________)

1. Do you smoke or Vape?

_____ No _____ cannabis only _____ cannabis+tobacco _____ tobacco only

1. Do you have any known upper body musculoskeletal problems?

_____ No ______ Yes (If so describe ________________________)

1. In the last 2 weeks have you had any of^#^ _____ NO _____ YES
   1. severe difficulty breathing (e.g., struggling for each breath, speaking in single words)
   2. severe chest pain
   3. having a very hard time waking up
   4. feeling confused
   5. lost consciousness
2. In the last 2 weeks have you had any of^#^ _____ NO _____ YES
   1. shortness of breath at rest
   2. inability to lie down because of difficulty breathing
   3. chronic health conditions that you are having difficulty managing because of your current respiratory illness
3. In the last 2 weeks have you had any of^#^ _____ NO _____ YES
   1. fever
   2. cough
   3. shortness of breath
   4. difficulty breathing
   5. sore throat
   6. runny nose
4. Are you currently experiencing any of ^#^ _____ NO _____ YES
   1. fever
   2. cough
   3. shortness of breath
   4. difficulty breathing
   5. sore throat
   6. runny nose

^#^ As extracted from <https://myhealth.alberta.ca/Journey/COVID-19/Pages/HWAssessTool.aspx#EmergencyCheck>
