# Supplementary material for: Empowering the willing: the feasibility of tele-mentored self-performed pleural ultrasound assessment for the surveillance of lung health
Source: Ultrasound J. 2022 Jan 3;14:2. doi: 10.1186/s13089-021-00250-6 (PMC9417136; doi:10.1186/s13089-021-00250-6)
Supplement: Supplementary file 5 — Additional file 4. Pleural Scoring System for COVID 19. [file 13089_2021_250_MOESM4_ESM.docx]

**Supplementary Material 4. Pleural Scoring System for COVID 19**

(from the Proposal for International Standardization of the Use of Lung Ultrasound for Patients With COVID‐19)(20)

The pleural Line will be scored as a 0,1,2, or 3 according to the following;

**Score 0 – Normal**

The pleural line is continuous and regular. Horizontal artifacts are present. These artifacts are generally referred to as A‐lines (See Figures 2-5). They are due to the high reflectivity of the normally aerated lung surface and characterize the visual representation of the multiple reflections happening between the US transducer and the lung surface itself


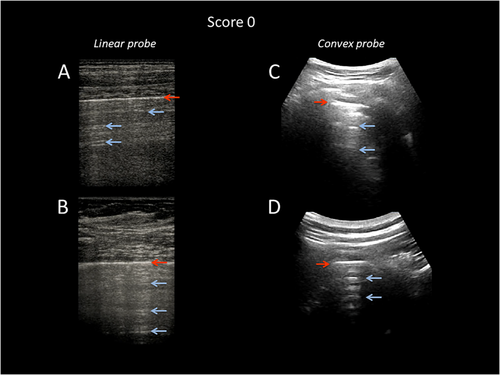


**Score 1 – Mildly Abnormal**

Score 1: The pleural line is indented. Below the indent, vertical areas of white are visible. These are due to local alterations in the acoustical properties of the lung, as, for example, the replacement of volumes previously occupied by air in favor of media that are acoustically much more similar to the intercostal tissue (water, blood, and tissue)


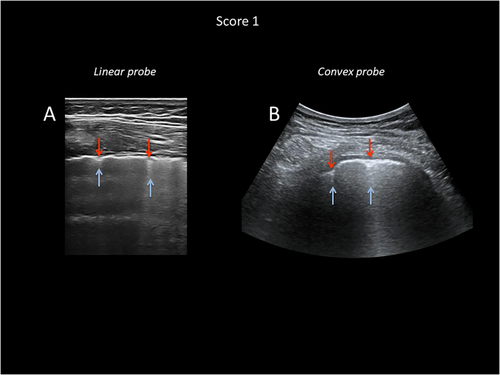


**Score 2 – Moderately Abnormal**

The pleural line is broken. Below the breaking point, small‐to‐large consolidated areas (darker areas) appear with associated areas of white below the consolidated area (white lung). The darkening of the consolidated areas signals the loss of aeration and the transition of these areas toward acoustic properties similar to soft tissue over the entire area represented by the consolidation itself. Beyond the consolidations, the appearance of areas of white lung signals the presence of areas not yet fully deaerated, where air inclusions are still present but embedded in tissue like material. This highly scattering environment can explain this peculiar pattern.


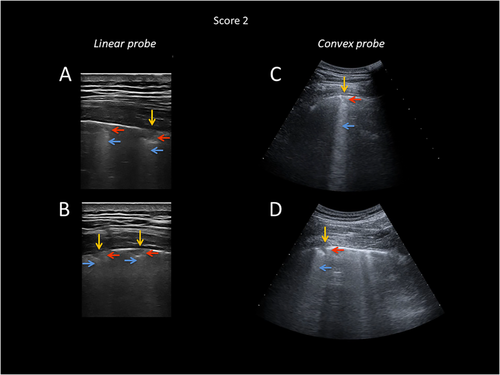


**Score 3 – Severely Abnormal**

The scanned area shows dense and largely extended white lung with or without larger consolidations

**
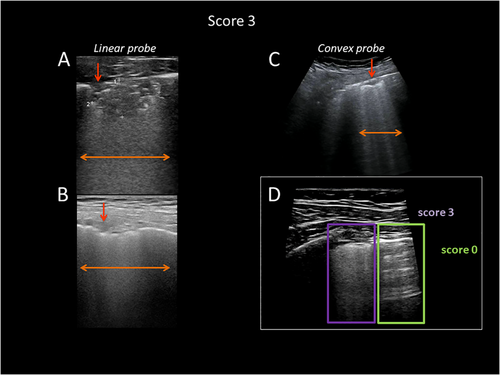
**
