# Supplementary material for: Empowering the willing: the feasibility of tele-mentored self-performed pleural ultrasound assessment for the surveillance of lung health
Source: Ultrasound J. 2022 Jan 3;14:2. doi: 10.1186/s13089-021-00250-6 (PMC9417136; doi:10.1186/s13089-021-00250-6)
Supplement: Supplementary file 6 — Additional file 5. Mentors and Blinded Reviewers Assessment of Pleural Lung Health Scoring. [file 13089_2021_250_MOESM5_ESM.docx]

**Supplementary Material 5. Mentors and Blinded Reviewers Assessment of Pleural Lung Health Scoring**

**Online Supplement __. Mentors and Blinded Reviewers Assessment of Pleural Lung Health Scoring**

**____________________________________________________________________________**

**^1^Anatomic ^2^Real-time ^3^Independant**

**Location Mentor Reviewers**

**(^4,5^Soldati Score 0) (^4,5^Soldati Score 0)**

**(^4,5^Soldati Score 1) (^4,5^Soldati Score 1)**

**(^4,5^Soldati Score 2) (^4,5^Soldati Score 2)**

**(^4,5^Soldati Score 3) (^4,5^Soldati Score 3)**

**[Comet-tails] [Comet-tails]**

**________________________________________________________________________**

**1) Right Upper Chest (96.3 % ) (88.9 % )**

**(3.7 % ) (11.1 % )**

**(0 % ) (0 % )**

**(0 % ) (0 % )**

**[ 0 ] [ 0 ]**

**2) Right Lower Chest (100% ) (84% )**

**(0% ) (16% )**

**(0% ) (0% )**

**(0% ) (0% )**

**[0 ] [0 ]**

**3) Left Upper Chest (92.6% ) (90.1% )**

**(3.7% ) (9.9% )**

**(3.7% ) (0% )**

**(0 % ) (0 )**

**[0 ] [0 ]**

**4) Left Lower Chest^6^ (100% ) (91.4% )**

**(0% ) (7.4% )**

**(0% ) (0% )**

**(0% ) (0% )**

**[0 ] [0 ]**

**5) Right Side Lower (92.6% ) (82.7% )**

**(3.7% ) (17.3% )**

**(3.7% ) (0% )**

**(0% ) (0% )**

**[0 ] [0 ]**

**6) Right Side Upper (100% ) (95.1% )**

**(0% ) (4.9% )**

**(0% ) (0%)**

**(0% ) (0% )**

**[0 ] [0 ]**

**7) Left Side Lower (100% ) (92.6% )**

**(0% ) (7.4% )**

**(0% ) (0% )**

**(0% ) (0% )**

**[0 ] [0 ]**

**8) Left Side Upper (92.6% ) (87.7% )**

**(3.7% ) (12.3% )**

**(0% ) (0% )**

**(0% ) (0% )**

**[0 ] [0 ]**

**9) Right Back Lower^6^ (88.5% ) (92.3% )**

**(3.8% ) (6.4% )**

**(3.8% ) (0% )**

**(0% ) (0% )**

**[0 ] [0 ]**

**10) Right Back Middle^6^ (90% ) (97.6% )**

**(5% ) (1.6% )**

**(5% ) (0% )**

**(0% ) (0% )**

**[0 ] [0 ]**

**11) Right Back Upper (87.5% ) (96.8% )**

**(12.5% ) (3.2% )**

**(0% ) (0% )**

**(0% ) (0% )**

**[0 ] [0 ]**

**12) Left Back Lower (96.2% ) (88.5% )**

**(3.8% ) (11.5% )**

**(0% ) (0% )**

**(0% ) (0% )**

**[0 ] [0 ]**

**15) Left Back Middle (94.1% ) (90.2% )**

**(5.9% ) (9.8% )**

**(0% ) (0% )**

**(0% ) (0% )**

**[0 ] [0 ]**

**16) Left Back Upper (100% ) (96.7% )**

**(0% ) (3.3% )**

**(0% ) (0% )**

**(0% ) (0% )**

**[0 ] [0 ]**

**_____________________________________________________________________________**

**Notes: ^1^Anatomic location is visually demonstrated in Figure 1.;**

**Legend: ^2^Real-time mentor evaluation during the interaction with the participant scoring adequacy, Soldati score, and mean number of comet tails demonstrated per location; ^3^Independant reviewer evaluation of recorded images of adequacy, Soldati score, and mean number of comet tails demonstrated per location; ^4^Soldati proposed score for pleural lung health(23); ^5^percentage of the exams that were possible to be performed and were of diagnostic quality; ^6^if a reviewer did not feel images were of diagnostic quality no scoring was done affecting the subsequent percentages..**
